# Customizing the electronic health record for delivery of pharmacogenetics

**DOI:** 10.1016/j.gimo.2023.100779

**Published:** 2023-03-08

**Authors:** Glenda Hoffecker, Lisa A. Varughese, Joseph Bleznuck, Jeffrey Landgraf, Collin Wollack, Jessica Chen, Marylyn D. Ritchie, Katherine L. Nathanson, Sony Tuteja

**Affiliations:** 1 Division of Translational Medicine and Human Genetics, Department of Medicine, Perelman School of Medicine, University of Pennsylvania, Philadelphia, PA; 2 Information Services Applications, Penn Medicine, University of Pennsylvania, Philadelphia, PA; 3 Department of Genetics, Perelman School of Medicine, University of Pennsylvania, Philadelphia, PA; 4 Abramson Cancer Center, Perelman School of Medicine, University of Pennsylvania, Philadelphia, PA

## Introduction

Pharmacogenomics (PGx) is the field of precision medicine that uses an individual’s genetic results to guide medication prescribing to optimize efficacy and prevent adverse drug reactions.^1^ To aid with PGx implementation, the Clinical Pharmacogenetics Implementation Consortium (CPIC) has published 26 evidence based, peer reviewed guidelines encompassing 21 pharmacogenes that impact more than 60 medications to facilitate the translation of PGx results into medication prescribing decisions.^2^ Most individuals will carry at least 1 PGx variant,^3^ which becomes important if they are prescribed a medication that has altered metabolism because of the variant they carry. Thus, integration of PGx results in the electronic health record (EHR) with the use of electronic clinical decision support (CDS) at the point of prescribing is a critical strategy for adoption and scaling of PGx into clinical care. A recent American College of Medical Genetics and Genomics (ACMG) technical standard outlines key components for pharmacogenomic result interpretation and reporting.^4^ We previously described our multidisciplinary effort to integrate discrete genomic data into the EHR.^5^^,^^6^ Here, we describe our process for customizing our EHR (Epic Systems Corporation, Verona, Wisconsin) called PennChart for PGx results while adhering to the best practices set out by the ACMG standards (Supplemental Table 1).

## Integration of Discrete PGx Results

To assist with the clinical interpretation of PGx results, ACMG recommends reporting genotype and metabolizer phenotypes, using CPIC’s activity score system for phenotype translation where pertinent.^4^ At Penn Medicine, PGx results can be found in a centralized location in PennChart called the “Precision Medicine” tab, a filtered list of laboratory orders only displaying genetic tests. Results generated by external laboratories, including commercial vendors (eg, OneOme, Minneapolis, MN), are electronically transmitted to PennChart, stored as a PDF file and discrete genotypes displayed in the form of diplotypes based on the Pharmacogene Variation (PharmVar) Consortium star allele definitions (eg, *DPYD* ∗2A/∗13).^7^ We have also integrated PGx results from a custom assay of the *DPYD* and *UGT1A1* genes using a laboratory-developed test with our partners at the Center for Applied Genomics (CAG) (Children’s Hospital of Philadelphia, Philadelphia, PA).^8^ Results are stored in PennChart as discrete diplotypes, phenotypes, and/or gene activity scores, as applicable. Activity scores are particularly important for drug–gene interactions (DGIs) for which CPIC’s recommendations vary for the same phenotype based on the activity score, as with the DPYD poor metabolizers with an activity score of 0 vs 0.5 when prescribing fluoropyrimidines.^9^ Translation tables, which were created by Epic using CPIC’s genotype–phenotype tables, are used in the background to interpret genotypes into phenotypes (and activity scores for *CYP2D6*, *CYP2C9*, and *DPYD*). These tables also automatically convert patient-specific genotypes into a simplified, user-friendly format called genomic indicators (Supplemental Figure 1). Genomic indicators can also be added manually by authorized individuals (eg, genetic counselors, pharmacogenetic pharmacists, and medical geneticists) for noninterfaced PGx results that are only available as a scanned PDF in the patient’s chart.

ACMG also recommends reporting medications that may be affected by PGx results and providing a list of resources to assist with therapeutic decision making.^4^ In PennChart, PGx genomic indicators can be viewed by health care providers in the snapshot view of each patient’s chart (front page of the chart) and display gene names and corresponding phenotype (eg, CYP2C19 poor metabolizer) with a brief description of the gene’s role in metabolizing and transporting medications in the body. Reference links to external educational resources (eg, Pharmacogenomics Knowledge Base [PharmGKB] gene summaries) are also included to deliver real-time education to clinicians who may not be familiar with PGx. We also created patient-friendly versions of the PGx genomic indicators that appear in the “My Genetic Profile” page of the patient’s myPennMedicine patient portal (ie, Epic’s MyChart). We use PGx genomic indicators in the logic of CDS alerts to call attention to DGIs at the time of prescribing.

## Creation of CDS Tools to Optimize Medication Prescribing at the Point of Care

In addition to implementing the recommendations outlined by the ACMG for PGx result reporting, we also provide an additional layer of result interpretation through CDS. CDS is an important strategy for dissemination of PGx knowledge to assist with therapeutic decision making across multiple clinical programs. A single pharmacogene can metabolize multiple medications so that the results of a PGx test ordered by a cardiology provider (eg, *CYP2C19* for clopidogrel nonresponse) will have ramifications for medications prescribed by primary care providers (eg, *CYP2C19* for escitalopram side effects). A well-designed CDS system is a key tenet in facilitating clinical translation of PGx results with minimal disruptions in existing workflows. Rule-based best practice alerts (BPAs) using available PGx evidence can help overcome barriers in implementation related to limited clinician knowledge. Interruptive CDS alerts appear as pop-up messages to communicate clinical recommendations based on a PGx result during medication order entry. Passive CDS consists of inline warnings that appear as informative banners within the medication ordering window and do not require action by the prescriber. Identification of DGIs via both interruptive and passive CDS ensures PGx information is accessible and applicable at the point of prescribing.

We primarily use passive alerts as the foundation of our PGx CDS, with interruptive CDS reserved for those DGIs for which CPIC recommends an alternative and/or use of the medication could cause serious adverse effects (eg, *CYP2C19*-clopidogrel). We currently have created 50 genomic indicators for 13 pharmacogenes (*DPYD*, *UGT1A1*, *CYP2B6*, *CYP2C9*, *CYP2C19*, *CYP2D6*, *CYP3A5*, *HLA-A*, *HLA-B*, *IFNL4*, *NUDT15*, *SLCO1B1*, and *TPMT*) and 211 medication-specific passive alerts providing real-time prescribing actions for 31 medications, prioritizing CPIC level A medications based on available guidelines. One gene has multiple genomic indicators to reflect gene activity (eg, CYP2C19 has 5 indicators—poor metabolizer, intermediate metabolizer, normal metabolizer, rapid metabolizer, and ultrarapid metabolizer). Triggering CDS at the genomic indicator level, as opposed to the discrete genotype or phenotype result components, allows for flexibility when new variants are discovered or when phenotype definitions change. However, in circumstances when a phenotype for a gene has differing therapeutic recommendations, custom rules in PennChart refer to the translation tables and genomic indicators to trigger the CDS. For example, it is recommended to avoid fluoropyrimidines in DPYD poor metabolizers with an activity score of 0, whereas a DPYD poor metabolizer with an activity score of 0.5 may receive a fluoropyrimidine at a reduced dose with early therapeutic drug monitoring in lieu of an alternative therapeutic agent. In these cases, our BPAs are triggered by relevant medication orders, the DPYD poor metabolizer genomic indicator and gene activity scores assigned to each diplotype in the translation table (Supplemental Figure 1).

Following the development of the CDS, extensive testing was performed by PGx subject matter experts in collaboration with pharmacy informatics team members to ensure BPAs appeared as intended. Using test patient charts with actionable PGx results, we reviewed each phenotype/activity score–medication order combination to validate the CDS build. EHR systems should be developed so that PGx-based recommendations can be added or revised as new clinical guidelines emerge from the growing knowledge base of DGIs.

## Dissemination of Best Practices for Integration of PGx

Standard vocabularies describing genetic results are critical for stakeholders to accurately generate and apply PGx results in the EHR for patient care. Using common terminology also facilitates the exchange of results with consistent variant interpretation between laboratories and institutions using EHRs.^10^ The supplemental tables from CPIC guidelines with suggested pre- and post-test alert language for each drug–gene pair are a useful resource for institutions seeking to create CDS for DGIs. As we customize our PGx build in PennChart, we will make available to the Epic community the genomic indicator language, CDS language for both passive and active alerts, tip sheets, and standard operative procedures for entering PGx data (https://www.med.upenn.edu/pgi). Uniform build across organizations that use Epic will eliminate confusion about interpretation of results for patients who receive care across multiple health care systems.

## Conclusion

We have made tremendous progress toward enabling a precision medicine approach to medication prescribing across our health system. The Epic genomics module provides the basic framework for the application of PGx-guided care, but substantial health system commitment and resources, including informatics and PGx specialists, were required to customize PGx result reporting and CDS. A dedicated team of PGx and informatics specialists meet weekly to plan, design, build, and test the content. Additional ongoing expertise and resources will be required to perform maintenance and quality control for the various components, eg, determining the optimal timing, frequency, and recipient of CDS alerts. Ongoing initiatives to enhance PGx implementation include (1) creating educational content for clinicians about PGx, (2) deploying the PGx build for hospitals on a separate instance of Epic, and (3) developing EHR tools that minimize clinician cognitive burden in pharmacotherapy decision making.

## Conflict of Interest

L.A.V is now an employee of Qral Group. J.C. is now an employee of the Walt Disney Company. All other authors declare no conflicts of interest.
