# Supplementary material for: Customizing the electronic health record for delivery of pharmacogenetics
Source: Genet Med Open. 2023 Mar 8;1(1):100779. doi: 10.1016/j.gimo.2023.100779 (PMC11613548; doi:10.1016/j.gimo.2023.100779)
Supplement: Supplemental Table 1 [file mmc1.docx]

Supplemental Table 1. Addressing the American College of Medical Genetics and Genomics (ACMG) technical standards for pharmacogenomic results interpretation and reporting.

| **ACMG recommendations** | **Addressed by EHR** | **Addressed by another method** |
| --- | --- | --- |
| 1. Report genotype and metabolizer phenotype (where applicable) | Discrete diplotypes & phenotype result stored in Epic’s variant masterfile and viewable in the “Precision Medicine” tab. | Listed on the results report sent by the testing laboratory. |
| 1. Provide a list of medications that may be affected by the identified genotype. | Passive or interruptive alerts at the point of prescribing. | Listed on the results report sent by the testing laboratory. |
| 1. Provide a generalized statement if an alternative therapy may be considered based on results. | Viewable in the brief description section of PGx genomic indicators. Provide alternative medications within interruptive BPAs. | Listed on the results report sent by the testing laboratory. |
| 1. Provide a list of resources that could inform actionable decisions (e.g., FDA tables, CPIC guidelines). | Provided as links in the PGx genomic indicators. | Listed on the results report sent by the testing laboratory. |
| 1. Clinical pharmacogenomic test reports should not provide patient-specific dosing. | Passive or interruptive alerts recommend dosage adjustments based on CPIC guidelines. | This information is not included in the results report sent by the testing laboratory. |
| 1. Include FDA therapeutic management recommendations in the clinical pharmacogenomic test report that currently have supportive evidence. | To address in future build. | Listed on the results report sent by the testing laboratory. |
| 1. Clearly state in the clinical pharmacogenomic test report that the accuracy of phenotypic prediction is dependent on the variants detected, as well as on the drug substrate if applicable. | To address in future build. | Listed on the results report sent by the testing laboratory. |
| 1. Clearly state in the clinical pharmacogenomic test report that drug–drug interactions can alter the metabolizer phenotype. | To address in future build. | Listed on the results report sent by the testing laboratory. |
| EHR: Electronic Health Record; CPIC: Clinical Pharmacogenetics Implementation Consortium; FDA: US Food and Drug Administration; PGx: Pharmacogenomic | | |
