# Supplementary material for: Customizing the electronic health record for delivery of pharmacogenetics
Source: Genet Med Open. 2023 Mar 8;1(1):100779. doi: 10.1016/j.gimo.2023.100779 (PMC11613548; doi:10.1016/j.gimo.2023.100779)
Supplement: Supplemental Figure 1 [file mmc2.pdf]

# Clinical Decision Support Logic Flow

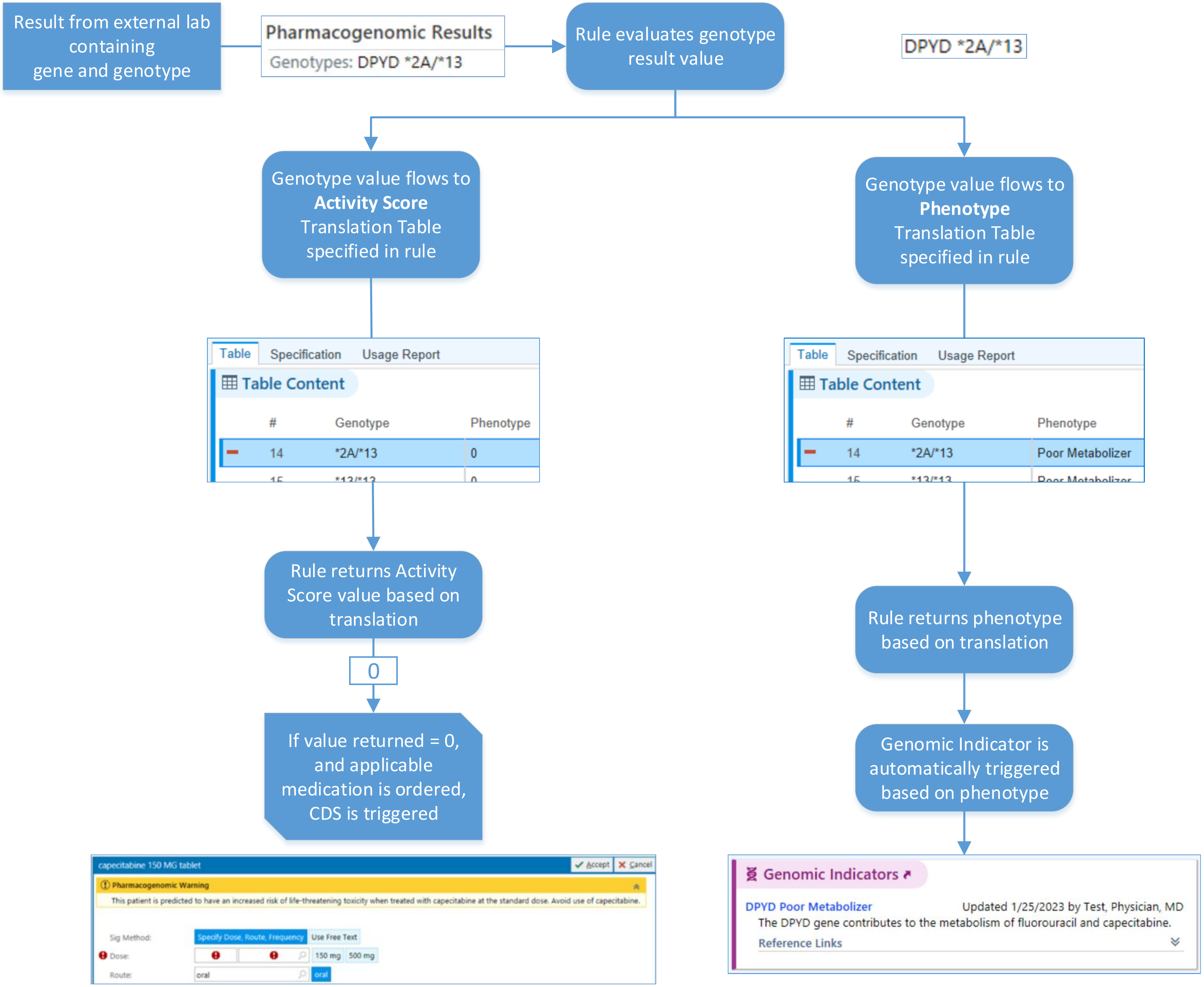


© 2021 Epic Systems Corporation
